# Are all digital hoarding behavior alike? Uncovering the pathways to digital hoarding behavior in the workplace

**DOI:** 10.3389/fpsyg.2026.1823038

**Published:** 2026-05-12

**Authors:** Zhao Dong, Mingxu Bao

**Affiliations:** 1 School of Management Science and Information Engineering, Business Big Data Research Center of Jilin Province, Jilin University of Finance and Economics, Changchun, China; 2 School of International Business, Jilin International Studies University, Changchun, China

**Keywords:** digital hoarding behavior, employee, fsQCA, information ecology theory, workplace

## Abstract

**Background:**

Individuals’ excessive preservation and accumulation of digital information may result in digital hoarding behavior. In the workplace, employees may engage in such behavior due to concerns about losing potentially useful information and its possible impact on task performance within the information ecology.

**Method:**

Drawing on information ecology theory, this study develops a configurational model to examine the antecedents of digital hoarding behavior in the workplace. Using fuzzy-set Qualitative Comparative Analysis (fsQCA), we empirically analyze survey data from 268 employees in China.

**Conclusion:**

The results reveal the complex and interdependent effects of the antecedent conditions on digital hoarding behavior, identifying three configurations that drive a high level of digital hoarding behavior in the workplace. Perceived ease of use emerges as a key condition that facilitates employees’ digital hoarding behavior. In contrast, a low level of digital hoarding behavior follows two distinct configurations, in which the absence of perceived usefulness and deficiency in AI adoption play important inhibitory roles.

**Discussion:**

This study extends the research perspective of digital hoarding behavior in the workplace and provides important practical guidance for managers seeking to improve digital information management and employee work efficiency.

## 1 Introduction

Advances in cloud storage and converged storage technologies have eliminated traditional limitations on personal storage capacity, fueling a widespread tendency toward excessive digital accumulation (Grover et al., 2022). This shift to online data storage has triggered a digital hoarding frenzy, characterized by the uncontrolled collection and retention of digital resources. While storing digital resources constitutes normal organizational practice, their excessive accumulation creates negative consequences for both organizations and employees (Chatterjee et al., 2021). In workplace, employees exhibit particularly pronounced and systematic digital hoarding behavior (Sillence et al., 2026), characterized by the excessive accumulation and retention of digital resources that often exceed operational requirements and storage practices.

As primary producers and users of organizational data, employees routinely interact with vast amounts of digital information (Bowen, 2016; Gao and Yu, 2025). Their professional responsibilities demand constant engagement with digital content from retrieving and archiving to synthesizing and disseminating data (Blount, 2011). These intensive operational needs, combined with the psychological ease of indefinite digital retention, create a self-reinforcing process where digital hoarding behavior becomes both a practical coping mechanism and an emergent risk to organizational efficiency. Organizational efficiency impacts include excessive server occupation (Sweeten et al., 2018), productivity degradation (McKellar et al., 2021), and network security threats (George, 2024), compounded by impaired decision-making latency from information overload. Concurrently, empirical studies demonstrate individual cognitive consequences from hoarding behavior, including increased cognitive load (Wu et al., 2024), impaired executive function (Vinoi et al., 2024b), cognitive depletion (Wang et al., 2023), and anxiety symptoms (Liu and Liu, 2025). Therefore, exploring the influencing factors of employees’ digital hoarding behavior can improve employees’ mental health (Sweeten et al., 2018; Thorpe et al., 2019), optimize personal data management practices, and help enterprises to improve data utilization efficiency (McKellar et al., 2024).

Scholars have paid attention to the influencing factors of digital hoarding behavior in social media contexts (Agarwal et al., 2024; Liu et al., 2023; Wang et al., 2023; Wu et al., 2024; Zaremohzzabieh et al., 2024; Bravo-Adasme et al., 2026). Although a few studies have examined digital hoarding in the workplace, they have primarily focused on its implications for job performance, responsibility, and control, rendering the antecedents of this behavior largely unexplored (Neave et al., 2019; Zhang and Chen, 2025). Social and economic development often overemphasizes the role of information technology in the workplace while ignoring individuals themselves (Braganza et al., 2021). Digital hoarding behavior is an irrational behavior caused by individuals’ concern about the impact of technology. It is influenced by various factors such as individual employees, environment, and technology (Sedera et al., 2022). Information ecology theory posits that within a defined informational ecosystem, information actors and their informational environments interact dynamically through information technologies, engaging in continuous cycles of information resources transmission and feedback mechanisms to establish equilibrium states that satisfy systemic needs (Nardi and O’Day, 2000; Yuan and Wang, 2022). The workplace constitutes an information ecosystem where information, actors, environment, and technology interacting to influence organizational dynamics (Chan et al., 2017; Jones, 2002). Employees’ digital hoarding behavior emerges as a complex phenomenon necessarily shaped by such information factors in the workplace. However, most existing studies on digital hoarding behavior have relied on conventional quantitative approaches, which tend to examine the independent influence of individual antecedents. Consequently, these studies provide limited insight into how employees interact with the information factors that affect digital hoarding behavior in the workplace.

This study explores the complex influence of multiple factors on digital hoarding behavior from an information ecology perspective. We focuses on the digital hoarding behavior of employee groups. While prior research has predominantly examined digital hoarding among students or general social media users (Wu et al., 2024; Zaremohzzabieh et al., 2024; Yue et al., 2025), this study redirects attention to the organizational context. Specifically, employees’ digital hoarding behavior in the workplace not only affects the individual work performance, but also may affect the management efficiency of the organization when these behaviors proliferate across teams and departments (McKellar et al., 2021). By examining digital hoarding among employee groups, this study extends existing research beyond students or social media contexts and highlights the significance of digital hoarding as a workplace information behavior.

To better explain employees’ digital hoarding in the workplace, the present study conceptualizes the workplace as an information ecosystem. Within this ecosystem, digital hoarding behavior is driven by the interaction of multiple informational conditions, including perceived usefulness, perceived ease of use, job responsibility, time pressure, and AI adoption. Drawing on information ecology theory, this study captures the multi-dimensional nature of workplace digital hoarding, thereby responding to calls for more systematic examinations of this phenomenon (Zhang and Chen, 2025). This theoretical perspective reveals how organizational information environments collectively shape hoarding behaviors and offers a more ecologically valid framework.

Building on this theoretical framework, this study employs fsQCA to explores the complex causal mechanism of digital hoarding behavior in the workplace. This application of fsQCA Method uncovers complex causal patterns neglected by conventional symmetric analyses. The findings not only align with the emerging recognition that digital hoarding involves complex interactions and synergistic effects among conditions (Zhang et al., 2025), but also provide empirical evidence for the causal asymmetry principle in organizational information behavior research.

To achieve the research objectives and goals, the remainder of the paper is structured as follows. This study systematically reviews the concept and characteristics of digital hoarding behavior across diverse contexts, analyzes its relationships with factors such as perceived usefulness, perceived ease of use, job responsibility, time pressure, and AI adoption, and develops a conceptual framework. Using the fsQCA method, the research identifies configurational pathways that influence digital hoarding behavior and discusses the findings to elucidate the systematic mechanisms underlying each path. Finally, the study addresses theoretical implications, managerial implications, limitations, and future research.

## 2 Literature review

### 2.1 Information ecology theory

Information ecology theory applies the concept of natural ecology to information science (Nardi and O’Day, 2000). Scholars have introduced the concept of the information ecosystem, proposing that within a specific environment, a system comprised of people, practices, technologies, and values constitutes an information ecosystem (Nardi and O’Day, 2000). Information ecology theory is applicable to causal interpretation of important, novel, and negative information behavior outcomes (Sussman and Siegal, 2003). It can make inferences about the attitudes or behaviors of individuals in the information environment, with the purpose of predicting and evaluating individual information activities (Yuan and Wang, 2022). The working situation of the organization is a positive ecosystem, and the generation and evolution of information factors can construct employees’ consciousness and behavior (Einola and Khoreva, 2023). There must be positive interactions between the constituent elements of a system (Naghshineh and Zardary, 2011).

Xu et al. (2023) state that an individual’s information behavior is the result of four factors: information, information actors, information environment, and information technology. The information provided at workplace has the characteristics of high importance, strong uncertainty, and high acquisition cost. These characteristics are closely related to improving the perceived usefulness and perceived ease of use before information interaction with users (Hess et al., 2014). Sweeten et al. (2018) confirmed that individuals’ perceived usefulness and perceived ease of use of digital resources are important inducements to enhance the desire to hoard and employees are unwilling to delete them. Employees are the subjects who consciously process information, and their behaviors will be influenced by individual emotions, motivations, or intentions. Sedera et al. (2022) believe that the internal motivation of employees’ digital hoarding behavior in the workplace mainly comes from their sense of job responsibility, because they need to retain data evidence to cope with future uncertainties. Time pressure from the information environment may make employees more likely to exhibit more digital hoarding behavior to avoid uncertainty (Wang et al., 2023). The adoption of AI makes it easier to obtain, save, call, and analyze data information. While this information technology empower employees to process vast data through intuitive commands, thereby enhancing decision-making efficiency (Sacramento et al., 2013), this very convenience paradoxically promote digital hoarding behavior as a risk mitigation strategy (Liu et al., 2023).

In this study, we have chosen perceived usefulness, perceived ease of use (information), job responsibility (information actors), time pressure (information environment), and AI adoption (information technology) in explaining digital hoarding behavior in the workplace. By dovetailing these factors, our model not only leverages information ecology theoretical frameworks but also addresses the complex interplay between information and digital hoarding behavior. This approach provides a comprehensive theoretical rationale for examining how different information factors influence digital hoarding behavior, strengthening the model’s theoretical grounding and applicability in the workplace.

### 2.2 Digital hoarding behavior

Digital hoarding behavior refers to individuals’ irrational and persistent accumulation of digital files (Neave et al., 2019). This compulsive behavior manifests through persistent difficulty discarding digital resources driven by distorted cognitive appraisals of perceived future value (McKellar et al., 2021), emotional attachment (Sweeten et al., 2018), and anxiety about data deletion (Wu et al., 2024). According to Sedera et al. (2022), digital hoarding behavior is a behavior of excessive acquisition of digital content and difficulty in deleting digital content, resulting in digital clutter. Wu et al. (2024) defined digital hoarding behavior in the social media context as the behavior of users who indiscriminately save data and are unwilling to delete data. Gao and Yu (2025) examined digital hoarding in the workplace, defining it as the employees’ tendency to continuously accumulate content on digital office platforms while being reluctant to delete it. Liu and Liu (2025) defined digital hoarding among college students as the persistent accumulation of and reluctance to delete digital materials, particularly academic resources.

Digital hoarding typically manifests as an unstructured accumulation without coherent themes, stemming from its inherently unstructured characteristics. As this behavior persists, it creates digital clutter and emotional distress, characterized by intense attachment to digital resources and persistent difficulty in deletion. Existing research on digital hoarding has predominantly centered on social media, workplace, and education. While individuals in these settings exhibit common traits such as emotional dependence and deletion difficulties, distinct behavioral patterns emerge across different user groups. Table 1 provides the analysis summary of digital hoarding behavior observed in these three contexts.

**Table 1:** The comparative analysis of digital hoarding behavior.

Category	Digital hoarding behavior		
	Social media	Workplace	Education
Actor	Social media users	Organizational employees	College students
Motivation	Affective and social requirements	Work demands and risk mitigation.	Academic requirements
Distinctiveness	Driven by Algorithms and Emotions	Driven by responsibility and pressure	Driven by achievements and values
Digital resources	Personalized and social resources	Work and task resources	Course materials and learning resources
Theory	Cognitive behavioral model; Attachment theory; Dual-factor theory and regret theory; Social comparison theory; Social cognitive theory; Perceived value theory;	Attachment theory; Conservation of Resources theory; Individual-environment interaction theory;	Attachment theory; Attentional overload theory; Individual-environment interaction theory;
Sources	Thorpe et al. (2019), Sedera et al. (2022), Wang et al. (2023), Vinoi et al. (2024a,b), Wu et al. (2024), Zhang and Chen (2025), and Sillence et al. (2026)	Sweeten et al. (2018), Neave et al. (2019), McKellar et al. (2024), and Gao and Yu (2025)	Liu et al. (2023), Zaremohzzabieh et al. (2024), and Liu and Liu (2025)

Employees’ digital hoarding behavior in the workplace constitutes a systematic pattern of excessive accumulating and retaining digital resources (Sweeten et al., 2018; Gao and Yu, 2025). This behavior initiated from dynamic information exchanges between individuals and their organizational ecosystem (McKellar et al., 2024). While initially driven by legitimate job requirements to preserve and organize digital resources, such behavior frequently evolve into excessive accumulation due to the inherent accessibility and storage capacity of digital platforms. Paradoxically, this digital hoarding behavior driven by responsibility and pressure ultimately generate substantial organizational management challenges (McKellar et al., 2021). Therefore, this study defines digital hoarding behavior as employees’ excessive acquisition of digital resources, including but not limited to electronic documents, multimedia files, email archives, software packages, and work-related digital artifacts. This behavior exceeds normal data collection processes through indiscriminate accumulation of digital resources, exhibiting unorganized management and deletion difficulties.

### 2.3 Perceived usefulness, perceived ease of use and digital hoarding behavior

Perceived usefulness and perceived ease of use play instrumental roles in explaining how employees’ perceptions of information value and susceptibility influence their digital hoarding behavior. Perceived usefulness refers to an individual’s subjective assessment of whether information or technology will enhance their performance or provide value, reflecting a cognitive evaluation of its practical benefits (Sussman and Siegal, 2003). The perceived usefulness of digital resources primarily manifests in how employees recognize and adapt to the intrinsic value of these resources in the workplace (Luo et al., 2024). The intrinsic value of digital resources includes both the immediate value perception and the future utility perception and estimation (Al-Emran et al., 2024). Whether employees choose to hoard a digital resource and ultimately take action depends on their perception and judgment of the usefulness of the resource. When employees think that a digital resource is useful to them now or in the future, they tend to choose to collect and save it. However, when information loses its value over time, employees exhibiting digital hoarding behavior demonstrate significant difficulty in discarding or deleting it.

Perceived ease of use refers to the degree of convenience or difficulty individuals perceive when acquiring, understanding, and using information (Davis, 1989). Hess et al.’s (2014) research suggests that the perception of information has a positive impact on employees’ attitudes and behavior towards information hoarding. Perceived ease of use cultivates individuals’ positive attitudes toward information retention, thereby reducing psychological barriers to discarding digital resources (Yao and Wang, 2024). This positive attitude, in turn, encourages employees to continuously store digital resources, ultimately developing into digital hoarding behavior. Studies supported the correlation between perceived ease of use and digital hoarding behavior. For instance, Vinoi et al. (2024a,b) confirmed that perceived ease of use significantly correlates directly and indirectly with digital hoarding. Understanding perceived usefulness and perceived ease of use is pivotal in this study as it provides insights into employees’ intrinsic assessments of digital resources in digital hoarding. It stands to reason that when employees believe perceived usefulness and perceived ease of use can relieve anxiety, they become more likely to engage in digital hoarding behavior.

### 2.4 Job responsibility and digital hoarding behavior

Job responsibility is the proactive commitment of employees to fulfill tasks with diligence, take ownership of both processes and outcomes, and contribute to organizational goals (Fox, 2009). As a form of positive work pressure that drives proactive engagement, job responsibility can enhance employees’ intrinsic motivation, thereby prompting greater effort investment in pursuit of achieving organizational goals (Jiandong et al., 2022). Digital resources, characterized by their convenience, durability, cost-effectiveness, replicability, and transferability, serve as an effective complement to human memory limitations (Sweeten et al., 2018). This implies that employees demonstrating strong responsibility orientations may preserve digital data as much as possible within their operational domains to safeguard informational completeness (Man Tang et al., 2022; McKellar et al., 2021). At the same time, digital information serves evidentiary purposes. Highly responsible employees systematically retain electronic work documents and communication records as verifiable evidence for potential future needs. The persistent and systematic accumulation of non-essential digital resources through timely and excessive preservation processes may ultimately cultivate employees’ digital hoarding behavior in the workplace.

### 2.5 Time pressure and digital hoarding behavior

Time pressure refers to the stress caused experienced by employees when confronting task demands that must be completed under constrained temporal conditions (Sacramento et al., 2013). Time pressure is a prevalent and challenging stress in the workplace. It can stimulate individuals’ action-oriented behavior (Gevers et al., 2006). In order to reduce the urgency and anxiety from time pressure, employees will expand the amount of information to cope with the high risk and high uncertainty in the task (Maruping et al., 2015). However, when processing digital information under constrained temporal conditions, employees often find it challenging to promptly evaluate its relevance and utility. This difficulty leads them to a preservation strategy as a dominant response to perceived scarcity threats, consequently accelerating information accumulation (Kocher and Sutter, 2006). Moreover, sustained adherence to the preservation strategy may induce cognitive dissonance regarding employees’ identity (Kühnel et al., 2012). This cognitive dissonance drives employees to chronically engage in preemptive information preservation, ultimately resulting in digital hoarding behavior.

### 2.6 AI adoption and digital hoarding behavior

AI adoption refers to the process in which employees in the workplace use AI technology (such as ChatGPT, intelligent robots, decision assistance, speech recognition) to achieve work goals and tasks (Kelley, 2022). While AI adoption optimizes work processes and boosts operational efficiency, it concurrently creates technological unemployment risks as employees may be substituted by AI (Frey and Osborne, 2017). In order to avoid being replaced, employees will apply AI technology to constantly search for new knowledge to improve their work skills (Einola and Khoreva, 2023). This enables employees to automatically acquire and analyze data with simple instructions, thereby predicting future trends and supporting data-driven decision-making (Cheng et al., 2023). The application of AI technology makes it easier to acquire, save, call and, analyze data information. Empirical studies demonstrate that the application of AI technology will affect employees’ cognition and behavior (Raisch and Krakowski, 2021). Therefore, employees’ technological displacement anxiety drives excessive data hoarding through AI technology, while the inherent convenience of AI perpetuates this digital hoarding behavior.

## 3 Research design

### 3.1 Research methodology

This study employs fuzzy-set Qualitative Comparative Analysis (fsQCA) to examine the configurational mechanisms underlying digital hoarding behavior in the workplace (Fiss, 2011; Pappas and Woodside, 2021). Unlike conventional approaches, which typically assume that a single linear model can explain behavior, fsQCA is well suited to capturing causal complexity by identifying multiple combinations of conditions that may lead to the same outcome (Skarmeas et al., 2014). It also enables researchers to assess how the presence or absence of specific conditions alters the role of other antecedents within a configuration (Geremew et al., 2024). Given that employees’ digital hoarding behavior in the workplace is likely to emerge from the interaction of cognitive, situational, and technological factors rather than from any single determinant, fsQCA provides an appropriate analytical lens for this study.

**Figure 1:**
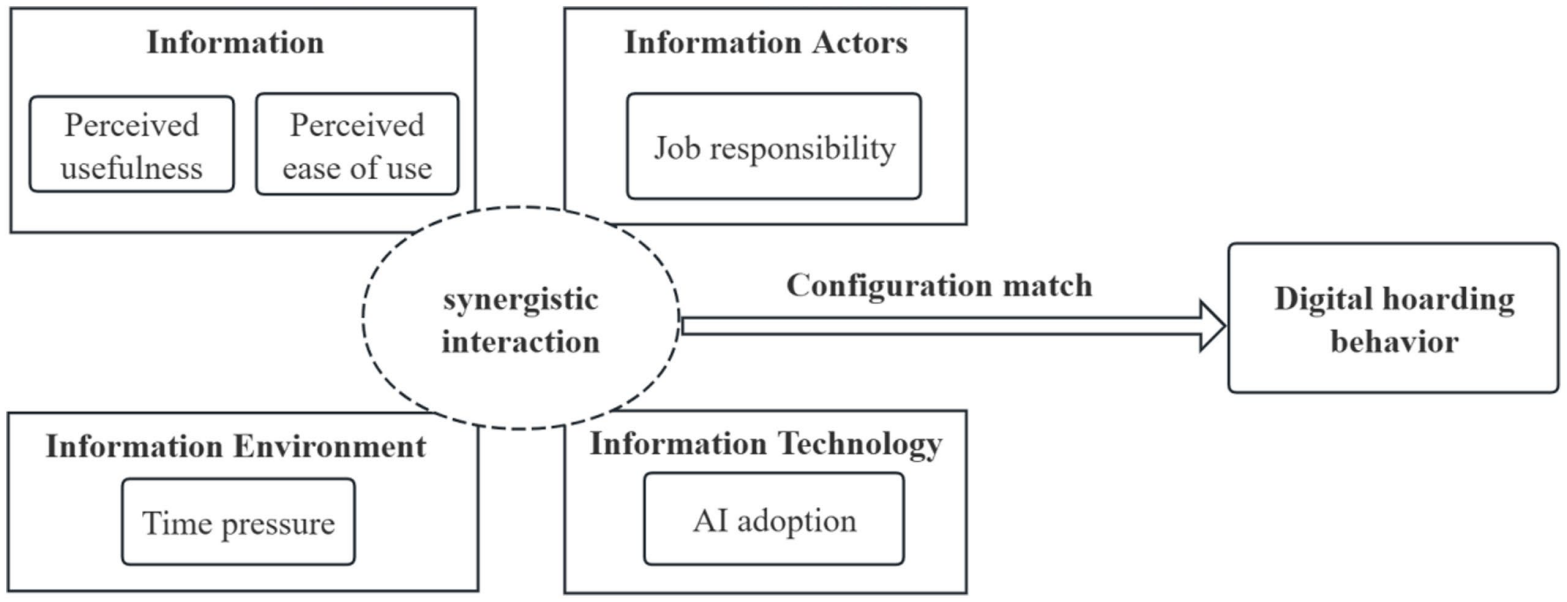
Conceptual model.

By adopting fsQCA, this study aims to capture the causal complexity of digital hoarding behavior in the workplace. Drawing on information ecology theory, we develop the conceptual framework to explain how multiple antecedent conditions jointly shape this behavior. In this framework, perceived usefulness and perceived ease of use represent the information dimension, job responsibility reflects the information actors dimension, time pressure captures the information environment dimension, and AI adoption represents the information technology dimension. Based on this perspective, workplace digital hoarding behavior is viewed not as the result of any single antecedent acting independently, but as an outcome produced by different combinations of interrelated conditions. The conceptual model is shown in Figure 1.

### 3.2 Conceptual measurement

This study utilized multiple scales from existing research, presenting each item as a statement to participants. To ascertain participants’ level of agreement or disagreement, we employed a 5-point Likert scale (1 = “strongly disagree” and 5 = “strongly agree”). The item validity of each variable’s scale was assessed by three scholars specializing in information behavior, with all measurement items derived from existing research. The evaluation procedure involved two main stages. First, panel members provided feedback on the wording, clarity, and comprehensibility of each item. Second, the panel examined potential problems among items and refined the construct measurement scheme to improve conceptual accuracy. These processes were designed to reduce semantic differences between items in international research. We recruited 20 MBA students as participants to test the scale, which allowed them to express their stance on each statement. The participants were invited to provide feedback on potential issues with the items. The scholars also determined whether any items were redundant and proposed improvements to ensure construct validity of the items.

Digital hoarding behavior was measured using the 10 item scale of Neave et al. (2019). The scale for measuring perceived usefulness and perceived ease of use was adapted from Davis’s (1989) research. The perceived usefulness scale and the perceived ease of use scale each contain 4 items. The Job responsibility scale was developed by Jiandong et al. (2022) with 3 items to measure sense of responsibility in the workplace. Time pressure scale was adapted from Maruping et al. (2015) with 4 items. The AI adoption scale, consisting of 7 items, was designed by Dong et al. (2025).

### 3.3 Data collection

This study employed a purposive sampling strategy because respondents were required to be currently employed and to have experience managing work-related digital information, as well as a basic understanding of digital hoarding behavior (Campbell et al., 2020). Data were collected through “Credamo” platform, a widely used online survey platform in China. The target respondents were employees working in different types of organizations and occupational positions. To improve the relevance of the sample, participant recruitment was conducted through screening procedures on the platform. Before accessing the full questionnaire, potential respondents were asked whether they were currently employed and whether they had experience handling digital information in the workplace. Only those who met these criteria were allowed to proceed to the main survey.

The survey questionnaire comprised four sections: study introduction, informed consent, measurement items, and demographic questions. Prior to participation, respondents were informed of the general purpose of the study, namely to examine employees’ digital hoarding behavior in the workplace. They were also informed that participation was entirely voluntary, that they could discontinue the survey at any time without penalty, and that their responses would be collected anonymously and used solely for academic research purposes. No personally identifiable information was collected. Participants received a small platform-based incentive upon completion of the survey.

The survey was administered online between March and May 2025. In total, 350 questionnaires were distributed and 284 were returned, yielding a response rate of 81.1%. After removing incomplete and invalid responses, 268 usable questionnaires were retained for analysis. The final sample included employees of different genders, age groups, educational backgrounds, firm types, and occupational positions, as summarized in Table 2.

**Table 2:** Demographic characteristics of respondents.

Characteristics	Category	Frequency	Percentage
Gender	Male	83	31.0%
	Female	185	69.0%
Age	≤30	128	47.8%
	31–40	103	38.4%
	41–50	22	8.2%
	>50	15	5.6%
Education	Less than High School	4	1.5%
	Associate	30	11.2%
	Bachelor	182	67.9%
	Master or Higher	52	19.4%
Firm Type	Government Agency	28	10.4%
	State-Owned Enterprise	66	24.6%
	Private Enterprise	160	59.7%
	Foreign-Invested Enterprise	14	5.3%
Position	Staff	126	47.0%
	Supervisor	65	24.3%
	Manager	63	23.5%
	Executive	14	5.2%

### 3.4 Common method variance test

To assess the presence of common method variance (CMV), Harman’s single-factor test was conducted. The results showed that the first factor accounted for 23.04% of the total variance, which is below the 40% threshold suggested by Podsakoff et al. (2003). Furthermore, all variance inflation factors (VIF) ranged from 1.131 to 1.268. These findings collectively indicate that common method bias is not a significant concern in this study.

### 3.5 Reliability and validity test

The measurement model was assessed for reliability and validity, with key metrics presented in Table 3. All factor loadings exceeded 0.7. The Cronbach’s *α* and composite reliability (CR) values for all constructs were above the 0.7 threshold, demonstrating adequate internal consistency. Furthermore, all average variance extracted (AVE) values surpassed 0.5, confirming convergent validity. These results collectively support the robustness of the measurement model.

**Table 3:** Measurement model assessment results.

Variable	Items	Loadings	Cronbach’s α	CR	AVE
Perceived usefulness	I trust that using digital resources improves my work efficiency.	0.789	0.838	0.842	0.573
	I trust that using digital resources enhances the quality of my work.	0.819			
	I believe that using digital resources makes my job easier.	0.771			
	I believe that using digital resources helps me complete tasks more quickly.	0.636			
Perceived ease of use	I find it easy to access digital resources.	0.778	0.823	0.823	0.538
	I find it straightforward to use digital resources.	0.728			
	I can quickly learn how to use digital resources.	0.738			
	I can easily understand the purpose of digital resources.	0.687			
Job responsibility	I work in the considerable responsibility.	0.737	0.793	0.802	0.577
	If the work at hand is not successful, I will have considerable responsibility.	0.854			
	My responsibilities range is very wide.	0.677			
Time pressure	We are often under a lot of pressure to complete our tasks on time.	0.802	0.885	0.887	0.664
	We are not afforded much time to complete our tasks.	0.893			
	The amount of time provided to complete our tasks is short.	0.847			
	Task durations are often short.	0.705			
AI adoption	I need AI to provide information resources to get the work done.	0.783	0.874	0.875	0.501
	I need AI to help me do my work.	0.702			
	I need to interact with the AI frequently at work.	0.697			
	I can use AI to do my work independently.	0.672			
	I see AI as my work partner.	0.750			
	My collaboration with AI at work is friendly.	0.697			
	AI supports my work.	0.644			
Digital hoarding behavior	I tend to accumulate digital files, even when they are not directly relevant to my job.	0.673	0.918	0.919	0.532
	I find it extremely difficult to delete old or unused files.	0.711			
	Deleting certain files would be like deleting a loved one.	0.813			
	If I delete certain files I feel apprehensive about it afterwards.	0.729			
	I strongly resist having to delete certain files.	0.774			
	I feel strongly that some files might be useful 1 day.	0.733			
	I lose track of how many digital files I possess.	0.748			
	Deleting certain files would be like losing part of myself.	0.703			
	Thinking about deleting certain files causes me some emotional discomfort.	0.673			
	At times I find it difficult to find certain files because I have so many.	0.722			

## 4 Research results

### 4.1 Variable calibration

Considering the research context and the nature of the questionnaire data, the study employed the direct calibration method to transform raw responses into fuzzy-set membership scores (Du et al., 2025). The dataset consists of 268 cases, each corresponding to one completed questionnaire and encompassing five antecedent conditions and one outcome variable. Following Fiss’s (2011) calibration procedure for 5-point Likert scales, the variable means were set as crossover points. Calibration anchors were defined using percentile values: full membership at the 95% percentile, full non-membership at the 5% percentile, and the crossover point at the 50% percentile. In instances where raw data coincided exactly with the crossover anchor, a minor adjustment (to 0.501 or 0.499) was applied to prevent case loss. All calibration procedures were performed using the fsQCA 3.0 software, which converted original variable values into fuzzy-set scores ranging from 0 to 1.

### 4.2 Analysis of necessary conditions

Analysis of necessary conditions in fsQCA examines whether a single antecedent condition is required for the outcome. This study evaluated five factors as potential necessary conditions for digital hoarding behavior, including perceived usefulness, perceived ease of use, job responsibility, time pressure, and AI adoption. In line with the causal asymmetry principle, the analysis was conducted separately for both high and low levels of digital hoarding behavior. Following the threshold suggested by Pappas and Woodside (2021), a condition was considered necessary only if both consistency and coverage exceeded 0.90. As shown in Table 4, none of the variables met this criterion, with all consistency values below 0.9. This indicates that neither high nor low levels of digital hoarding behavior among employees are driven by any single factor alone, but rather emerge from configurations of multiple concurrent conditions.

**Table 4:** Results of necessary conditions analysis.

Conditional variables	A high level of digital hoarding behavior		A low level of digital hoarding behavior	
	Consistency	Coverage	Consistency	Coverage
Perceived usefulness	0.619445	0.715029	0.549388	0.521040
~Perceived usefulness	0.585066	0.612444	0.699523	0.601636
Perceived ease of use	0.709309	0.780849	0.575265	0.520319
~Perceived ease of use	0.564266	0.617876	0.757706	0.681692
Job responsibility	0.762130	0.753099	0.658655	0.534751
~Job responsibility	0.529173	0.653601	0.695891	0.706198
Time pressure	0.686437	0.719466	0.619186	0.533213
~Time pressure	0.554642	0.639338	0.674233	0.663856
AI adoption	0.699549	0.762685	0.600051	0.537508
~AI adoption	0.575794	0.636659	0.735071	0.667789

### 4.3 Results of the configurational analysis

Appropriate threshold settings are critical to ensuring the robustness and explanatory of configuration analysis in fsQCA. We established thresholds for both case frequency and consistency. The case frequency threshold was set at 2, meaning that only configurations observed in at least 2 cases within the sample were retained. This criterion covers approximately 75% of the valid cases, ensuring that the results are both representative and statistically robust. In terms of consistency, a solution consistency threshold of 0.8 and a PRI consistency threshold of 0.7 were adopted. These thresholds help effectively distinguish subset relationships and ensure that the identified configurations possess sufficient explanatory power for the outcome variable (Du et al., 2025). Collectively, these thresholds ensure the methodological and substantive validity of the analytical outcomes.

To present the configurational pathways clearly, this study adopts typical representation method proposed by Ragin (2008). In this method, “
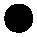
” denotes the presence of a core condition, “
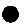
” the presence of a peripheral condition, “
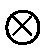
” the absence of a core condition, and “
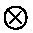
” the absence of a peripheral condition. As shown in Table 5, the analysis yielded three configurations sufficient for a high level of digital hoarding behavior (H1, H2, H3) and two for a low level (NH1, NH2). All configurations exhibited consistency scores above the 0.8 acceptability threshold. The overall solution coverage for a high level of digital hoarding behavior is 0.548. This indicates that the identified configurations account for a substantial proportion of the variance in the outcome.

**Table 5:** Configurational pathways of digital hoarding behavior.

Conditions	A high level of digital hoarding behavior			A low level of digital hoarding behavior	
	H1	H2	H3	NH1	NH2
Perceived usefulness		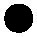	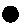	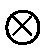	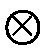
Perceived ease of use	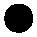	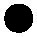	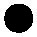	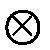	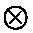
Job responsibility	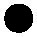	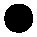		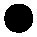	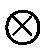
Time pressure	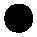		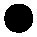	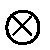	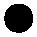
AI adoption		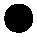	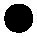	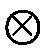	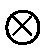
Consistency	0.888467	0.911039	0.904466	0.912031	0.903874
Raw coverage	0.446969	0.318946	0.391063	0.283641	0.336049
Unique coverage	0.142508	0.014485	0.086602	0.062849	0.115257
Overall consistency	0.880453			0.877728	
Overall coverage	0.548055			0.398897	

### 4.4 Configurational pathways for a high level of digital hoarding behavior

The configurational pathways leading to a high level of digital hoarding behavior were categorized into three models according to the characteristics of the covered cases: Time-motivated accumulated model, Cognition-Technology facilitated model, and Externally induced model.

Configuration path H1 (Time-motivated accumulated model) demonstrates that perceived ease of use, job responsibility, and time pressure are the core conditions. This path shows that the combination of high perceived ease of use, high job responsibility, and high time pressure is sufficient for a high level of digital hoarding behavior, regardless of perceived usefulness and AI adoption.

Configuration path H2 (Cognition-Technology facilitated model) indicates that perceived usefulness, perceived ease of use, job responsibility, and AI adoption are the core conditions. This path indicates that, regardless of time pressure, the combination of high job responsibility, high perceived usefulness, high perceived ease of use, and high AI adoption is sufficient for a high level of digital hoarding behavior.

Configuration path H3 (Externally induced model) indicates that perceived ease of use, time pressure, and AI adoption are core conditions, while perceived usefulness is a peripheral condition. This path indicates that the combination of high perceived ease of use, high time pressure, and high AI adoption, together with the peripheral presence of perceived usefulness, is sufficient for a high level of digital hoarding behavior.

### 4.5 Configurational pathways for a low level of digital hoarding behavior

Based on the analysis of core insufficient conditions, two distinct configurational pathways (NH1, NH2) were found to lead to a low level of digital hoarding behavior. A consistently lack of perceived usefulness and lack of AI adoption as core absent conditions across both paths. Low perceived usefulness and low AI adoption appear as core absent conditions across both paths.

Low perceived ease of use also appears in both configurations, although its role differs across the two paths. Specifically, it acts as a core absent condition in NH1 and a peripheral absent condition in NH2. These results indicate that low perceived usefulness, low perceived ease of use, and low AI adoption jointly contribute to the formation of low digital hoarding behavior, although their configurational roles vary across the two paths.

The two configurations further differ in terms of job responsibility and time pressure. In NH1, job responsibility is present as a core condition, whereas time pressure is a core absent condition. In NH2, job responsibility is a core absent condition, whereas time pressure is present as a core condition. These differences indicate that low digital hoarding behavior may arise through multiple configurations rather than through a single causal condition.

### 4.6 Robustness testing

According to the robustness testing methods of existing literature (Du et al., 2025), we conducted two tests to assess the stability of the fsQCA results. First, the original consistency threshold was raised from 0.80 to 0.85, and the empirical analysis was reperformed. Second, the case frequency threshold was increased from 2 to 3, followed by a re-examination of the configuration solutions. In both tests, the resulting configurations remained unchanged, with no notable variations in consistency or coverage metrics. These outcomes confirm that the findings of this study are methodologically robust.

## 5 Discussion

### 5.1 Configurational explanations for a high level of digital hoarding behavior

Perceived usefulness and perceived ease of use are increasingly recognized as important factors driving the formation of digital hoarding behavior (Vinoi et al., 2024b). The fsQCA results indicate that perceived ease of use emerges across all three configurations associated with a high level of digital hoarding behavior, underscoring its foundational role in shaping digital hoarding behavior among employees in the workplace. This suggests that the digitalization in the workplace substantially reduces employees’ cost and benefit evaluation of information acquisition. Cognitive resources are allocated more to information acquisition, leading employees to store digital items almost automatically and develop digital hoarding behavior. This is also consistent with the inference of the cognitive behavioural model regarding the origins of digital hoarding (Vinoi et al., 2024b).

This result stands in notable contrast to the classical Technology Acceptance Model (Davis, 1989), which traditionally positions perceived usefulness as the primary antecedent of technology related behavior. Our findings suggest an asymmetry in which perceived ease of use serves as a core condition, whereas perceived usefulness acts as a peripheral but supportive factor. The Technology Acceptance Model was originally built on the assumption that users make conscious and rational decisions when adopting new technology, emphasizing the dominant role of perceived usefulness in driving behavioral intention. However, digital hoarding behavior is essentially a form of information-saving behavior characterized by low cognitive involvement and a high degree of automaticity (Sillence et al., 2026). It is not based on a careful evaluation of the value of digital resources, but rather stems from the avoidance of immediate operational costs (Vinoi et al., 2024b). Perceived ease of use directly increases behavioral tendency for acquiring and storing information, making the saving of digital resources an automatic response, thereby representing a departure from the traditional logic of the Technology Acceptance Model. This means that when behavior becomes increasingly automatic, perceived ease of use may replace perceived usefulness as a more critical antecedent.

From an information ecology perspective (Nardi and O’Day, 2000), this pattern shows an imbalance in the workplace information environment. When the effort required to store information becomes very low, employees may no longer carefully decide what should be kept and what should be removed. As a result, the information environment becomes permissive of accumulation, and employees within it adapt accordingly. This is consistent with the research findings that, in AI supported or high pressure work situations, employees tend to rely on simple and efficient actions rather than carefully judging the actual value of information (Wang et al., 2022). Our results support this view by showing that perceived ease of use, rather than perceived usefulness, plays a more central role in leading to a high level of digital hoarding behavior in the workplace.

Configuration path H1, the “Time-motivated accumulated model” indicates that high levels of perceived ease of use, job responsibility, and time pressure are conducive to high digital hoarding behavior. Previous research has suggested that time pressure may suppress hoarding behavior by forcing individuals to prioritize tasks and make selective decisions (Kocher and Sutter, 2006), which seems to contradict our findings. However, drawing on the scarcity mindset, employees under time pressure are more likely to perceive resource scarcity and therefore adopt preemptive accumulation as a coping strategy (Maruping et al., 2015). Job responsibility amplifies this effect. Employees with strong job responsibility are motivated to retain materials just in case, interpreting comprehensive information retention as professional diligence. Perceived ease of use removes the final barrier to high digital hoarding behavior. This configuration thus represents a Time pressure driven hoarding pathway, which has been insufficiently examined in prior digital hoarding research.

Configuration path H2, the “Cognition-Technology facilitated model” indicates that high levels of perceived usefulness, perceived ease of use, job responsibility, and AI adoption are conducive to high digital hoarding behavior. This suggests that employees who regard digital resources as both easy to use and valuable are further driven by job responsibility and the integration of AI adoption. Rather than substituting for information storage as one might expect, AI tools appear to expand the perceived utility of accumulated data. Employees may recognize that AI technology can extract value from large datasets that would be unmanageable manually, thereby redefining digital hoarding as a form of data capital investment. This motivates them to continuously store and seldom discard digital resources, thereby sustaining high digital hoarding behavior. This finding extends prior work by Vinoi et al. (2024a,b), who identified perceived usefulness as a driver of digital hoarding, by demonstrating that the mechanism is further conditioned by AI adoption and job responsibility.

Configuration path H3, the “Externally induced model” indicates that high levels of perceived ease of use and job responsibility, along with AI adoption while perceived usefulness remains peripheral, are conducive to high digital hoarding behavior. This pathway is distinct from both H1 and H2. The absence of high job responsibility as a core condition suggests that this configuration captures a more externally induced form of digital hoarding. This may reflect a situation where employees working under significant time pressure and supported by AI technology tend to are unable to engage in deliberate information evaluation, bypassing selective deletion and accelerating the accumulation of digital resources. From an information ecology perspective, when the information technology component of the ecosystem operates faster than the human actor can deliberate, the system undermines the autonomy of individual decision making and induces passive hoarding behaviors. This aligns with the research by Méndez-Suárez et al. (2025), which suggests that information overload caused by AI may impair individual decision making ability, leading to passive coping strategies such as indiscriminate information hoarding.

### 5.2 Configurational explanations for a low level of digital hoarding behavior

The fsQCA results revealed that there is not a single factor leading to a low level of digital hoarding behavior. Instead, multiple configurations that include the presence and absence of certain conditions can lead to this desirable outcome. Table 4 indicates that there are two configurations that lead to a low level of digital hoarding behavior, namely “Perceived value absence model” and “Motivational deficiency model”. Low perceived usefulness and low AI adoption are two core non-identification conditions across both paths. These conditions revealing the conditions that inhibit digital hoarding behavior are not simply the negations of those that promote it, a pattern consistent with the asymmetric causality principle fundamental to configurational analysis (Fiss, 2011; Ragin, 2008).

The joint absence of perceived usefulness and AI adoption transform the original information ecosystem in which neither the cognitive valuation of information nor the AI technological for automated accumulation provides impetus for digital hoarding behavior. These findings suggest that when digital resources are perceived as low in value and unsupported by AI tools, employees are more likely to inhibit digital hoarding behavior. This is consistent with the perspective of information ecology theory that the mismatch between information and information technology can lead to changes in individual cognition and behavior (Nardi and O’Day, 2000).

Configuration path NH1 indicates that low perceived usefulness, low perceived ease of use, high job responsibility, low time pressure, and low AI adoption are associated with low digital hoarding behavior. The presence of high job responsibility might be expected to drive hoarding, as it does in H1 and H2, yet here it fails to produce that outcome. This suggests that job responsibility is not an insufficient driver of digital hoarding behavior. Its effect is contingent on the availability of perceived value and technology. Employees who consider digital resources neither useful nor easy to use tend to refrain from extensive hoarding, even when they exhibit a strong sense of job responsibility. Meanwhile, the absence of AI tools further restricts automated accumulation. This type of employees reflects conscious and effortful evaluation rather than automated saving, thereby suppressing digital hoarding behavior. This finding suggests that an important intervention method corroborates Bravo-Adasme et al.’s (2026) research findings that increasing the perceived cognitive cost may be more likely to inhibit digital hoarding behavior even among highly motivated employees.

Configuration path NH2 indicates that low perceived usefulness, low perceived ease of use, low job responsibility, high time pressure, and low AI adoption lead to low digital hoarding behavior. High time pressure was shown in H1 and H3 to promote digital hoarding behavior, yet here it appears to suppress it. The critical differentiating factor is the absence of perceived usefulness, job responsibility, and AI adoption. In the case of configuration path NH2, the lack of perceived usefulness is compounded by significant time pressure. Under high time pressure, the absence of both perceived value and AI support leads employees to disregard irrelevant digital resources, thereby further inhibiting digital hoarding behavior. This finding explains the apparent contradiction and contributes to a more nuanced understanding of the role of time pressure in information ecology and behavior. It also reveals that time pressure is essentially configuration and context dependent, thereby answering the call for enriching the research context of time pressure in digital hoarding (Zhang et al., 2025).

## 6 Theoretical and management implications

### 6.1 Theoretical implications

First, this study extends the digital hoarding literature by moving beyond general media users and consumer contexts to examine employees in the workplace. Although digital hoarding has received increasing scholarly attention in social media and education (Wang et al., 2023; Agarwal et al., 2024; Zhang and Chen, 2025; Bravo-Adasme et al., 2026), its formation in organizational environments remains insufficiently examined (Gao and Yu, 2025). Employees, as a significant group in the workplace, have individual needs, motivations, and external environments that are fundamentally distinct from users in daily social media settings (Wolf, 2013). The factors influencing employees’ digital hoarding behavior in the workplace cannot be fully inferred from findings derived from social or personal digital environments. By examining how perceived usefulness, perceived ease of use, job responsibility, time pressure, and AI adoption jointly shape employees’ digital hoarding behavior, this study identifies specific set of antecedent conditions for digital hoarding behavior in the workplace. In this sense, our findings extend the scope of digital hoarding research and deepen understanding of employee information behavior in organizational settings.

Second, this study contributes to digital hoarding behavior research by revealing its configurational and asymmetric causal structure. Prior research examining antecedents of digital hoarding has largely employed traditional approaches, presuming a linear relationship in which the presence of a factor amplifies hoarding and its absence diminishes it (Yue et al., 2025; Bravo-Adasme et al., 2026). Our fsQCA results show that high and low digital hoarding behavior arise from different combinations of conditions. Specifically, a high level of digital hoarding is driven by three distinct pathways, which we name “Time-motivated accumulated model,” “Cognition-Technology facilitated model,” and “Externally induced model.” In contrast, there are two configurations that lead to a low level of digital hoarding behavior, namely “Perceived value absence model” and “Motivational deficiency model.” Perceived ease of use serve as a fundamental factor for a high level of digital hoarding behavior, while for a low level of digital hoarding behavior, the joint absence of perceived usefulness and AI adoption forms the core basis of the outcome. These findings demonstrate that the conditions leading to high digital hoarding are not simply the reverse of those leading to low digital hoarding, thereby confirming the causal asymmetry of digital hoarding behavior. It indicates that management plans formulated for a high level of digital hoarding behavior might not inevitably influence a low level of digital hoarding behavior. This study therefore advances the literature by offering a configurational framework that captures the causal complexity and asymmetry of digital hoarding behavior in the workplace.

Third, from an information ecology perspective, our findings identify a boundary condition for perceived ease of use and offer empirical support for the research of employees’ digital hoarding behavior in the workplace. The Technology Acceptance Model emphasizes perceived usefulness as the primary antecedent of information behavior (Davis, 1989). However, our findings show that perceived ease of use appears as a core condition across all high level digital hoarding behavior configurations, while perceived usefulness plays a peripheral and supportive role. This suggests that when digital hoarding behavior becomes low in cognitive involvement and highly automatic, the explanatory priority of perceived usefulness may weaken, and perceived ease of use may become the more critical antecedent. This provides a boundary condition for applying the Technology Acceptance Model to information behavior. From an information ecology perspective (Nardi and O’Day, 2000), the finding further shows that digital hoarding is shaped by the interaction among perceived usefulness, perceived ease of use, job responsibility, time pressure, and AI adoption. By showing that these factors impact digital hoarding behavior, our study not only supports the perception that information change leads to hoarding behavior but also expands it by integrating the nuanced roles of environment and technology in shaping these behaviors. Our results enhance the theoretical applicability of information ecology theory in explaining individual information behaviors, providing a deeper understanding of the influencing digital hoarding behavior in the workplace.

### 6.2 Managerial implications

The results of this study have several implications for employees and managers. First, as both producers and consumers of digital resources, employees handle large amounts of digital data. Excessive accumulation of digital resources not only reduces personal productivity but may also lead to disorganized information, triggering psychological issues such as anxiety and exhaustion. In severe cases, it can develop into typical information disorders, including digital hoarding, information anxiety, and digital addiction (Sweeten et al., 2018). Therefore, managers should help employees establish healthier digital work routines by setting clearer norms for saving, classifying, reviewing, and deleting digital resources. At the same time, they should reduce employees’ uncertainty about information loss through organizational guidance and support.

Second, managers should prioritize developing employees’ capabilities in digital resource collection, identification, and application. Research indicates that low perceived usefulness is an important factor driving high digital hoarding behavior. Employees need to enhance their ability to assess the value of digital information, as overestimating content worthiness may exacerbate hoarding tendencies (Vinoi et al., 2024a). At the same time, perceived ease of use is identified as a core condition across all high digital hoarding behavior configurations, indicating that convenient storage systems may unintentionally encourage automatic and excessive hoarding behavior. Therefore, it is crucial for managers to effectively reduce excessive digital hoarding behavior by improving employees’ ability to evaluate information value and establish a system review mechanism.

Third, managers should actively promote the application and adoption of AI technology within organizations. The adoption of AI technology has a significant impact on adjusting employees’ digital hoarding behavior. In both configurations of the low level of digital hoarding behavior, AI adoption is a core deficiency across all pathways. The findings indicate that when AI technology becomes an indispensable part of the work, employees are more likely to utilize these advanced tools for efficient information processing rather than hoarding digital resources. Therefore, organizations should provide accessible AI tools, relevant training, and clear usage support to help employees manage digital resources more efficiently and reduce digital hoarding behavior.

## 7 Limitations and future directions

This study provides meaningful insights into the informational antecedents of digital hoarding in workplace settings, though several limitations should be acknowledged alongside promising avenues for future research. First, the use of cross-sectional data limits causal inference regarding the evolution of digital hoarding behavior. Longitudinal research would provide a stronger basis for understanding how digital hoarding behavior emerges, persists, or changes across different stages of work and technology use. Second, this study does not sufficiently differentiate among the types and characteristics of digital resources accumulated by employees. Digital hoarding may involve various forms of digital resources in the workplace. Future studies could incorporate more fine-grained measures of digital resource characteristics, or usage-based measures may offer an understanding of how specific forms of digital resources contribute to digital hoarding behavior. Third, while the proposed theoretical model is substantiated in the Chinese context, its generalizability across cultures remains to be established. Digital hoarding behavior may be influenced by local culture, laws, regulations, and organizational management, and information retention. Future research could examine whether the configurational patterns identified in this study remain stable across different national contexts.

## 8 Conclusion

Situated within enterprises’ digital transformation, this research addresses pressing management challenges associated with employees’ digital hoarding behavior. Drawing on information ecology theory and applying fsQCA, this study shows that workplace digital hoarding is not driven by a single antecedent, but by the combined effects of multiple informational conditions. The results indicate that digital hoarding behavior is influenced by the joint influence of information, information actors, the information environment, and information technology. This conclusion aligns with the configurational perspective on digital hoarding proposed by Vinoi et al. (2024a, 2024b), and further reinforces the understanding that digital hoarding behavior in the workplace is indeed motivated by a combination of interrelated information factors.

This study identifies three configurational pathways leading to high levels of digital hoarding behavior among employees, which are named the “Time-motivated accumulated model,” “Cognition-Technology facilitated model,” and “Externally induced model.” Perceived ease of use is a common core condition leading to high levels of digital hoarding behavior, suggesting that when digital resources are easy to store, employees may be more likely to accumulate them excessively. This finding stands in contrast to the Technology Acceptance Model that perceived usefulness is the more critical determinant of information behavior. Moreover, perceived usefulness, job responsibility, time pressure, and AI adoption exhibit varying roles as contributing factors in different configurations. The findings demonstrate that no single condition suffices to explain digital hoarding behavior. Instead, the influence of antecedent variables differs markedly across configurations. For instance, perceived usefulness, perceived ease of use and AI adoption contribute to high digital hoarding behavior in paths H2 and H3, but such a combination is absent in H1. Similarly, variables such as job responsibility and time pressure also exhibit contingent effects. Thus, this finding confirms that the impact of different variable combinations on employees’ digital hoarding behavior exhibits both complexity and synergistic effects.

Our findings reveal two distinct pathways associated with low levels of employees’ digital hoarding behavior, namely the “perceived value absence model” and the “motivational deficiency model.” Low perceived usefulness combined with limited AI adoption emerges as the core conditions inhibiting digital hoarding behavior. This result suggests that when employees perceive digital resources as having limited practical value and lack sufficient AI support for information management, they will reduce their digital hoarding behavior in the workplace. This evidence align with the view of Sweeten et al. (2018) that ineffective value transmission and technology adaptation diminish digital hoarding practices. It also indicates that the antecedent configurations associated with low digital hoarding are not simply the reverse of those leading to high digital hoarding, thereby confirming the causal asymmetry of this behavior. In conclusion, from an information ecology perspective, this study uncovers the configurational pathways underlying both high and low levels of digital hoarding behavior in the workplace. It advances understanding of digital hoarding behavior as a complex information practice in organizational settings. At the same time, these findings provide practical guidance for enterprises seeking to reduce employees’ digital hoarding behavior and enhance information management efficiency.

## Data Availability

The raw data supporting the conclusions of this article will be made available by the authors, without undue reservation.

## References

[ref1] Agarwal R. Mehrotra A. Pant M. K. Alzeiby E. A. Vishnoi S. K. (2024). Digital photo hoarding in online retail context. An in-depth qualitative investigation of retail consumers. J. Retail. Consum. Serv. 78:103729. doi: 10.1016/j.jretconser.2024.103729

[ref2] Al-Emran M. Al-Sharafi M. A. Foroughi B. Iranmanesh M. Alsharida R. A. Al-Qaysi N. . (2024). Evaluating the barriers affecting cybersecurity behavior in the Metaverse using PLS-SEM and fuzzy sets (fsQCA). Comput. Human Behav. 159:108315. doi: 10.1016/j.chb.2024.108315

[ref3] Blount Y. (2011). Employee management and service provision: a conceptual framework. Inf. Technol. People 24, 134–157. doi: 10.1108/09593841111137331

[ref4] Bowen D. E. (2016). The changing role of employees in service theory and practice: an interdisciplinary view. Hum. Resour. Manag. Rev. 26, 4–13. doi: 10.1016/j.hrmr.2015.09.002

[ref5] Braganza A. Chen W. Canhoto A. Sap S. (2021). Productive employment and decent work: the impact of AI adoption on psychological contracts, job engagement and employee trust. J. Bus. Res. 131, 485–494. doi: 10.1016/j.jbusres.2020.08.018, 32836565 PMC7434459

[ref6] Bravo-Adasme N. Cataldo A. Grandón E. E. Riquelme J. Rayo Y. Reyes C. (2026). From the cluttered room to the full hard drive: the relationship between hoarding disorder and digital hoarding. Behav. Sci. 16:429. doi: 10.3390/bs16030429, 41898090 PMC13023865

[ref7] Campbell S. Greenwood M. Prior S. Shearer T. Walkem K. Young S. . (2020). Purposive sampling: complex or simple? Research case examples. J. Res. Nurs. 25, 652–661. doi: 10.1177/1744987120927206, 34394687 PMC7932468

[ref8] Chan E. S. Hon A. H. Okumus F. Chan W. (2017). An empirical study of environmental practices and employee ecological behavior in the hotel industry. J. Hosp. Tour. Res. 41, 585–608. doi: 10.1177/1096348014550873

[ref9] Chatterjee S. Rana N. P. Dwivedi Y. K. Baabdullah A. M. (2021). Understanding AI adoption in manufacturing and production firms using an integrated TAM-TOE model. Technol. Forecast. Soc. Change 170:120880. doi: 10.1016/j.techfore.2021.120880

[ref10] Cheng B. Lin H. Kong Y. (2023). Challenge or hindrance? How and when organizational artificial intelligence adoption influences employee job crafting. J. Bus. Res. 164:113987. doi: 10.1016/j.jbusres.2023.113987

[ref11] Davis F. D. (1989). Perceived usefulness, perceived ease of use, and user acceptance of information technology. MIS Q. 13, 319–340. doi: 10.2307/249008

[ref12] Dong X. Tian Y. He M. Wang T. (2025). When knowledge workers meet AI? The double-edged sword effects of AI adoption on innovative work behavior. J. Knowl. Manag. 29, 113–147. doi: 10.1108/JKM-02-2024-0222

[ref13] Du Y. Liu Q. Kim P. H. Li J. (2025). Riding the waves of change: using qualitative comparative analysis to analyze complex growth patterns in entrepreneurship. Entrep. Theory Pract. 49, 312–353. doi: 10.1177/10422587241249330

[ref14] Einola K. Khoreva V. (2023). Best friend or broken tool? Exploring the co-existence of humans and artificial intelligence in the workplace ecosystem. Hum. Resour. Manag. 62, 117–135. doi: 10.1002/hrm.22147

[ref15] Fiss P. C. (2011). Building better causal theories: a fuzzy set approach to typologies in organization research. Acad. Manag. J. 54, 393–420. doi: 10.5465/amj.2011.60263120

[ref16] Fox J. T. (2009). Firm-size wage gaps, job responsibility, and hierarchical matching. J. Labor Econ. 27, 83–126. doi: 10.1086/597428

[ref17] Frey C. B. Osborne M. A. (2017). The future of employment: how susceptible are jobs to computerisation? Technol. Forecast. Soc. Change. 114, 254–280. doi: 10.1016/j.techfore.2016.08.019

[ref18] Gao C. Yu C. (2025). Exploring the effect of digital hoarding in the workplace on employee work performance. Front. Psychol. 16:1198825. doi: 10.3389/fpsyg.2025.1198825, 41488956 PMC12756427

[ref19] George A. S. (2024). Digital hoarding: the rising environmental and personal costs of information overload. Partners Univ. Multidiscip. Res. J. 1, 51–67. doi: 10.5281/zenodo.12802575

[ref20] Geremew Y. M. Huang W.-J. Hung K. (2024). Fuzzy-set qualitative comparative analysis as a mixed-method and analysis technique: a comprehensive systematic review. J. Travel Res. 63, 3–26. doi: 10.1177/00472875231168619

[ref21] Gevers J. M. Rutte C. G. Van Eerde W. (2006). Meeting deadlines in work groups: implicit and explicit mechanisms. Appl. Psychol. 55, 52–72. doi: 10.1111/j.1464-0597.2006.00228.x

[ref22] Grover P. Kar A. K. Dwivedi Y. K. (2022). Understanding artificial intelligence adoption in operations management: insights from the review of academic literature and social media discussions. Ann. Oper. Res. 308, 177–213. doi: 10.1007/s10479-020-03683-9

[ref23] Hess T. J. McNab A. L. Basoglu K. A. (2014). Reliability generalization of perceived ease of use, perceived usefulness, and behavioral intentions. MIS Q. 38, 1–28. doi: 10.25300/MISQ/2014/38.1.01

[ref24] Jiandong S. Fan X. Haitian L. (2022). How do high-performance work systems affect work fatigue: the mediating effect of job responsibility and role overload. PLoS One 17:e0269452. doi: 10.1371/journal.pone.0269452, 35793365 PMC9258864

[ref25] Jones S. (2002). Employee rights, employee responsibilities and knowledge sharing in intelligent organization. Employ. Responsib. Rights J. 14, 69–78. doi: 10.1023/A:1021119503782

[ref26] Kelley S. (2022). Employee perceptions of the effective adoption of AI principles. J. Bus. Ethics 178, 871–893. doi: 10.1007/s10551-022-05051-y, 35818389 PMC9259894

[ref27] Kocher M. G. Sutter M. (2006). Time is money—time pressure, incentives, and the quality of decision-making. J. Econ. Behav. Organ. 61, 375–392. doi: 10.1016/j.jebo.2004.11.013

[ref28] Kühnel J. Sonnentag S. Bledow R. (2012). Resources and time pressure as day-level antecedents of work engagement. J. Occup. Organ. Psychol. 85, 181–198. doi: 10.1111/j.2044-8325.2011.02022.x

[ref29] Liu Y. Chi X. Xin X. (2023). Storing, not Reading: investigating the link between upward social comparison via social media and digital hoarding behavior in Chinese youth. Psychol. Res. Behav. Manag. 16, 5209–5224. doi: 10.2147/PRBM.S441859, 38152591 PMC10752025

[ref30] Liu Y. Liu Y. (2025). Hoarding knowledge or hoarding stress? Investigating the link between digital hoarding and cognitive failures among Chinese college students. Front. Psychol. 15:1518860. doi: 10.3389/fpsyg.2024.1518860, 39949973 PMC11821920

[ref31] Luo J. Ahmad S. F. Alyaemeni A. Ou Y. Irshad M. Alyafi-Alzahri R. . (2024). Role of perceived ease of use, usefulness, and financial strength on the adoption of health information systems: the moderating role of hospital size. Humanit. Soc. Sci. Commun. 11, 1–12. doi: 10.1057/s41599-024-02976-9

[ref32] Man Tang P. Koopman J. McClean S. T. Zhang J. H. Li C. H. De Cremer D. . (2022). When conscientious employees meet intelligent machines: an integrative approach inspired by complementarity theory and role theory. Acad. Manag. J. 65, 1019–1054. doi: 10.5465/amj.2020.1516

[ref33] Maruping L. M. Venkatesh V. Thatcher S. M. Patel P. C. (2015). Folding under pressure or rising to the occasion? Perceived time pressure and the moderating role of team temporal leadership. Acad. Manag. J. 58, 1313–1333. doi: 10.5465/amj.2012.0468

[ref34] McKellar K. Sillence E. Neave N. Briggs P. (2021). There is more than one type of hoarder: collecting, managing and hoarding digital data in the workplace. Interact. Comput. 32, 209–220. doi: 10.1093/iwc/iwaa015

[ref35] McKellar K. Sillence E. Neave N. Briggs P. (2024). Digital accumulation behaviours and information management in the workplace: exploring the tensions between digital data hoarding, organisational culture and policy. Behav. Inf. Technol. 43, 1206–1218. doi: 10.1080/0144929X.2023.2205970

[ref36] Méndez-Suárez M. Ćukušić M. Ninčević-Pašalić I. (2025). AI FoMO (fear of missing out) in the workplace. Technol. Soc. 84:103052. doi: 10.1016/j.techsoc.2025.103052

[ref37] Naghshineh N. Zardary S. (2011). Information ecology as a mind tool for repurposing of educational social networks. Procedia. Soc. Behav. Sci. 15, 3640–3643. doi: 10.1016/j.sbspro.2011.04.348

[ref38] Nardi B. A. O’Day V. (2000). Information Ecologies: Using Technology with Heart. Cambridge: Mit Press.

[ref39] Neave N. Briggs P. McKellar K. Sillence E. (2019). Digital hoarding behaviours: measurement and evaluation. Comput. Human Behav. 96, 72–77. doi: 10.1016/j.chb.2019.01.037

[ref40] Pappas I. O. Woodside A. G. (2021). Fuzzy-set qualitative comparative analysis (fsQCA): guidelines for research practice in information systems and marketing. Int. J. Inf. Manag. 58:102310. doi: 10.1016/j.ijinfomgt.2021.102310

[ref41] Podsakoff P. M. MacKenzie S. B. Lee J.-Y. Podsakoff N. P. (2003). Common method biases in behavioral research: a critical review of the literature and recommended remedies. J. Appl. Psychol. 88, 879–903. doi: 10.1037/0021-9010.88.5.879, 14516251

[ref1101] Ragin C. C. (2008). Redesigning social inquiry: Fuzzy sets and beyond. Berkeley, CA: University of California Press.

[ref42] Raisch S. Krakowski S. (2021). Artificial intelligence and management: the automation–augmentation paradox. Acad. Manag. Rev. 46, 192–210. doi: 10.5465/amr.2018.0072

[ref43] Sacramento C. A. Fay D. West M. A. (2013). Workplace duties or opportunities? Challenge stressors, regulatory focus, and creativity. Organ. Behav. Hum. Decis. Process. 121, 141–157. doi: 10.1016/j.obhdp.2013.01.008

[ref44] Sedera D. Lokuge S. Grover V. (2022). Modern-day hoarding: a model for understanding and measuring digital hoarding. Inf. Manag. 59:103700. doi: 10.1016/j.im.2022.103700

[ref45] Sillence E. Dawson J. A. Brown R. D. McKellar K. Neave N. (2026). Digital hoarding and personal use digital data. Hum. Comput. Interact. 41, 6–25. doi: 10.1080/07370024.2023.2293001

[ref46] Skarmeas D. Leonidou C. N. Saridakis C. (2014). Examining the role of CSR skepticism using fuzzy-set qualitative comparative analysis. J. Bus. Res. 67, 1796–1805. doi: 10.1016/j.jbusres.2013.12.010

[ref47] Sussman S. W. Siegal W. S. (2003). Informational influence in organizations: an integrated approach to knowledge adoption. Inf. Syst. Res. 14, 47–65. doi: 10.1287/isre.14.1.47.14767

[ref48] Sweeten G. Sillence E. Neave N. (2018). Digital hoarding behaviours: underlying motivations and potential negative consequences. Comput. Human Behav. 85, 54–60. doi: 10.1016/j.chb.2018.03.031

[ref49] Thorpe S. Bolster A. Neave N. (2019). Exploring aspects of the cognitive behavioural model of physical hoarding in relation to digital hoarding behaviours. Digit. Health. 5:2055207619882172. doi: 10.1177/2055207619882172, 31636918 PMC6785915

[ref50] Vinoi N. Shankar A. Khalil A. Mehrotra A. Kumar J. (2024a). Holding on to your memories: factors influencing social media hoarding behaviour. J. Retail. Consum. Serv. 76:103617. doi: 10.1016/j.jretconser.2023.103617

[ref51] Vinoi N. Shankar A. Mehrotra A. Kumar J. Azad N. (2024b). Enablers and inhibitors of digital hoarding behaviour. An application of dual-factor theory and regret theory. J. Retail. Consum. Serv. 77:103645. doi: 10.1016/j.jretconser.2023.103645

[ref52] Wang H. Miao P. Jia H. Lai K. (2023). The dark side of upward social comparison for social media users: an investigation of fear of missing out and digital hoarding behavior. Soc. Media Soc. 9:20563051221150420. doi: 10.1177/20563051221150420

[ref53] Wang H. Zhang H. Chen Z. Zhu J. Zhang Y. (2022). Influence of artificial intelligence and robotics awareness on employee creativity in the hotel industry. Front. Psychol. 13:834160. doi: 10.3389/fpsyg.2022.834160, 35300168 PMC8922015

[ref54] Wolf J. (2013). Improving the sustainable development of firms: the role of employees. Bus. Strateg. Environ. 22, 92–108. doi: 10.1002/bse.1731

[ref55] Wu D. Zhao Y. C. Wang X. Song S. Lian J. (2024). Digital hoarding in everyday hedonic social media use: the roles of fear of missing out (FoMO) and social media affordances. Int. J. Hum. Comput. 40, 5399–5414. doi: 10.1080/10447318.2023.2233139

[ref56] Xu X. J. Xu L. Z. Wang X. L. (2023). An ecological evaluation and empirical study of mobile payment information ecosystem. Inf. Sci. 41, 182–190. doi: 10.13833/j.issn.1007-7634.2023.04.022

[ref57] Yao N. Wang Q. (2024). Factors influencing pre-service special education teachers’ intention toward AI in education: digital literacy, teacher self-efficacy, perceived ease of use, and perceived usefulness. Heliyon. 10:e34894. doi: 10.1016/j.heliyon.2024.e34894, 39149079 PMC11325385

[ref58] Yuan X. Wang C. (2022). Research on the formation mechanism of information cocoon and individual differences among researchers based on information ecology theory. Front. Psychol. 13:1055798. doi: 10.3389/fpsyg.2022.1055798, 36605281 PMC9809296

[ref59] Yue Z. Zhong L. Zhang W. Zheng X. (2025). A study on the influence mechanism of emotional interaction and consumer digital hoarding in agricultural live social E-commerce. J. Theor. Appl. Electron. Commer. Res. 20, 1–17. doi: 10.3390/jtaer20040331

[ref60] Zaremohzzabieh Z. Abdullah H. Ahrari S. Abdullah R. Md Nor S. M. (2024). Exploration of vulnerability factors of digital hoarding behavior among university students and the moderating role of maladaptive perfectionism. Digit. Health. 10:20552076241226962. doi: 10.1177/20552076241226962, 38298527 PMC10829496

[ref61] Zhang X. Chen X. (2025). What drives digital hoarding? Understanding the impact of perceived value and the mediating role of security on digital hoarding behavior. Curr. Psychol. 44, 1333–1346. doi: 10.1007/s12144-024-07268-8

[ref62] Zhang C. Li J. Hao Y. (2025). Why do “digital hamsters” hoard but never consume? Configurational pathways and influencing mechanisms of digital hoarding behaviour among Chinese generation Z. Behav. Sci. 15:1575. doi: 10.3390/bs15111575, 41301376 PMC12649117

